# Effect of Ordinary Portland Cement on Mechanical Properties and Microstructures of Metakaolin-Based Geopolymers

**DOI:** 10.3390/ma15249007

**Published:** 2022-12-16

**Authors:** Renhui Gao, Wei Yang, Zhenhua Duan, Hui Liu, Qi Deng, Minqi Hua

**Affiliations:** 1 Department of Civil Engineering, Changzhou University, Changzhou 213164, China; 2 Zhongshan Dongjun Concrete Co., Ltd., Zhongshan 528403, China; 3 Department of Structural Engineering, Tongji University, Shanghai 200092, China; 4 School of Civil Engineering and Architecture, Wuhan University of Technology, Wuhan 430070, China

**Keywords:** cement, mechanical properties, microstructures, metakaolin, geopolymers

## Abstract

Geopolymers have been considered a sustainable alternative to ordinary Portland cement (CEM I) for its lower embodied carbon and ability to make use of industrial by-products. Additionally, its excellent engineering properties of high strength, low permeability, good chemical resistance, and excellent fire resistance also strike a chord in the minds of researchers. The goal of this study is to clarify the effect of calcium sources on the mechanical properties and microstructures of the geopolymers. CEM I was chosen as the sole calcium source, while metakaolin was used as the source material. Five distinct geopolymers were prepared, having various ratio of CEM I: 0%, 5%, 10%, 20%, and 30%. The alkali-activator was a mixture of 12 M sodium hydroxide (NaOH) and sodium silicate (Na_2_SiO_3_), utilizing compressive strength and flexural strength to evaluate the changes of the geopolymers’ mechanical properties. SEM, XRD, and FTIR were used to examine microscopic features, evaluate internal morphology, and analyze changes in components of the geopolymers containing different amounts of CEM I. The experimental results indicated that the optimal incorporation of CEM I was 5%. Under this dosage, the compressive strength and flexural strength of the geopolymers can reach 71.1 MPa and 6.75 MPa, respectively. With the incorporation of CEM I, the heat released by cement hydration can accelerate the geopolymerization reaction between silica-alumina materials and alkaline solutions. Additionally, the coexistence of N-A-S-H gel from components of an aluminosilicate mix and C-S-H gel from the CEM I promoted a more densified microstructure of the geopolymers and improved the geopolymer’s strength. However, as the amount of CEM I in the mixture increased, the geopolymer matrix was unable to provide enough water for the CEM I to hydrate, which prevented excessive CEM I from forming hydration products, weakening the workability of the matrix and eventually hindering the development of geopolymer strength.

## 1. Introduction

In recent years, the increased awareness of environmental problems and the increased need for sustainable development have made the recycling of waste a priority [1]. According to reports, 1.55 billion tons of general industrial solid waste (non-hazardous solid waste generated in production and life) and 46.40 million tons of industrial hazardous waste (solid waste with hazardous characteristics such as corrosiveness, combustibility, toxicity, etc.) were produced in 200 large and medium cities in China, which led to serious environmental issues [2]. In addition, the aggravation of the greenhouse effect has raised people’s concerns about energy conservation and emission reduction. Conventional ordinary Portland cement is one of the main contributors to global warming, emitting 2.2 Gt carbon dioxide (CO_2_) annually [3,4], which accounts for 5–7% of the total worldwide emissions [5,6,7]. It was reported that about 17% of the total emissions are related to the construction and building industry [8]. This will contribute to global warming and seriously hinder the achievement of carbon peak and carbon neutrality [9]. Thus, building materials desperately need new development directions.

Geopolymers are an effective solution for recycling industrial solid waste and by-products (fly ash, ground granulated blast furnace slag, metakaolin, etc.) that has received widespread attention. As an emerging binder, they were initially developed and coined by Davidovits in 1972 [10]. Geopolymers are inorganic gelling substances made from aluminosilicate source materials that have been chemically activated by a strongly alkaline solution and are preferably cured at high temperatures [11]. Additionally, the silicon-oxygen tetrahedron and aluminum-oxygen tetrahedron’s amorphous three-dimensional network structure have given geopolymers exceptional engineering qualities such as high strength, high-temperature resistance, low permeability, and high durability, among others [12,13,14]. In comparison with CEM I, geopolymers not only have better mechanical properties and durability but also a 60–80% lower carbon footprint [15]. Additionally, Heath et al. [16] found that the application of alkali-activated materials can reduce global warming by 30–40%. Undoubtedly, geopolymers have been proposed as a more environmentally friendly alternative to CEM I.

With regard to geopolymers, a great deal of research has been done by scholars. Yang et al. [17] used metakaolin (calcium-free), high-calcium fly ash, and low-calcium fly ash as precursors, examining the geopolymer concrete’s ability to resist corrosion caused by sulfuric acid when made with various precursors. They found that more gypsum crystals are generated in the high-calcium fly-ash-based specimen, sealing the specimen’s pores and prevent further acid solution erosion and giving it greater acid resistance than the other specimens. Saengsuree et al. [18] investigated how Portland cement was added to a high-calcium fly ash geopolymer to change its characteristics during the curing process. The findings showed that water curing and vapor-proof membrane curing increased CEM I hydration, which increased compressive strength. Temuujin et al. [19] examined the effects of calcium compounds on the physical properties of fly-ash-based geopolymer paste cured at ambient and elevated temperatures and demonstrated that the addition of calcium compounds as a fly ash substitute would improve the mechanical properties for the ambient temperature cured samples. Cho et al. [20] also concluded that the absence of calcium in the geopolymer mix may contribute to a longer geopolymer setting time and sometimes slow the strength gain and increased shrinkage at an early age. Thus, the calcium content of geopolymer binding material has a great impact on the durability and physical properties of the geopolymers. However, the existing studies have not been involved in the effect of calcium source on the mechanical properties and microstructures of calcium-free-based geopolymers.

In this paper, CEM I was chosen as the sole calcium source and kaolinite was prepared as a calcium-free geopolymer binding material by dehydroxylation followed by calcination thermal activation. Due to the stacked layers configuration, the metakaolin had two types (metakaolinite and metadiskaolinte). According to the density functional theory, the metakaolinite was at a water chemical potential of −2.67 eV and metadiskaolinite was at a water chemical potential of −2.319 eV. Moreover, the metakaolinite was formed by heating Al_12_Si_12_O_39_(OH)_6_ to 655 °C and releasing the remaining three hydroxyl groups. Additionally, to reach complete dihydroxylation, Al_12_Si_12_O_33_(OH)_18_ was heated to 547 °C for the final production of metadiskaolinite. The metakaolinite was chosen as the binding material in this study [21]. To clarify the effect of the calcium source on the mechanical properties and microstructures of the metakaolin-based geopolymer, the metakaolin was replaced by CEM I at rates of 0%, 5%, 10%, 20%, and 30% respectively. The mechanical properties of the geopolymers were evaluated by compressive strength and flexural strength. Additionally, SEM, XRD, and FTIR were used to examine the microscopic properties of the geopolymers containing various contents of CEM I in order to investigate the internal morphology and assess the component changes.

## 2. Materials and Methods

### 2.1. Raw Materials

Metakaolin was purchased from Gongyi, Henan Province, and P·O 42.5 Portland cement (CEM I) was produced by Jiangsu Yangzi Cement Co., Ltd. (Changzhou, China). Table 1 shows the physical properties of metakaolin and CEM I. Additionally, their chemical compositions measured by x-ray fluorescence (XRF) are given in Table 2. The reagent of sodium hydroxide (NaOH, AR) was purchased from Sinopharm Chemical Reagent Co., Ltd., Shanghai, China. Sodium silicate liquid (Na_2_SiO_3_ with 29.9 wt% SiO_2_, 13.75 wt% Na_2_O, and 56.35 wt% H_2_O) and distilled water were used in the experiments.

### 2.2. Mix Proportions and Preparation of Specimens

The binding materials and alkali activators were mixed in the light of the mixing proportions presented in Table 3. The alkaline activator was a 1:1.5 (by mass) mixture of sodium hydroxide (NaOH) solution and sodium silicate (Na_2_SiO_3_) solution. The NaOH solution with concentration of 12 M was prepared 24 h in advance. Specimens were prepared with alkali-activator and metakaolin under a ratio (liquid/solid) of 0.8.

**Figure 1:**
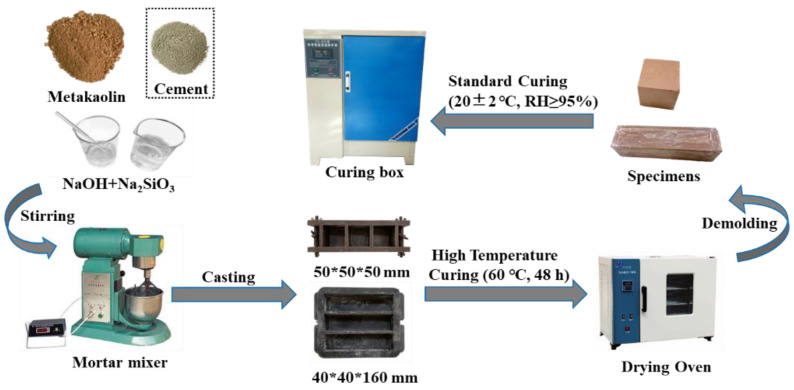
Preparation and curing process of geopolymers.

Figure 1 revealed the preparation and curing process of the geopolymers. Firstly, all of the NaOH solution and binding materials were mixed and stirred for 90 s. Then, the Na_2_SiO_3_ solution was added and stirred for another 90 s. The mixes were then put into molds measuring 50 mm × 50 mm × 50 mm and 40 mm × 40 mm × 160 mm, respectively, and vibrated for 60 s to remove air bubbles. Next, the filled specimens were heat-cured at 60 °C for 48 h while being covered with hermetic bags to prevent excessive moisture loss. After reaching the heat-curing age, the specimens that had cooled for 1 h (a delay of about 1 h between demolding and heat curing was beneficial in improving the properties of the geopolymers [22,23]) were demolded and preserved in the standard curing room (20 ± 2 °C, relative humidity (RH) ≥ 95%) until the standard curing age of 7 d. It should be noted that the binding materials consisted of a mixture of metakaolin and CEM I, and the substitution rate of CEM I was 0%, 5%, 10%, 20%, and 30% respectively. Hence, the specimens were marked as G_0_, G_5_, G_10_, G_20,_ and G_30_.

### 2.3. Testing Methods

#### 2.3.1. Mechanical Properties Test

The compressive strength and flexural strength of the geopolymers replaced by CEM I with different substitution rates were tested by an electro-hydraulic servo universal testing machine (YNS 300) in accordance with the Chinese Standard GB/T 50081-2019 [24]. The value of compressive strength and flexural strength is the average of three specimens.

#### 2.3.2. Microscopic Performance Test

Using a scanning electron microscope (SEM, SUPRA55, Zeiss, Jena, Germany) at an accelerating voltage of 15 kV, the morphologies of the geopolymers replaced by CEM I at various substitution rates were examined.

By using an X-ray diffractometer (XRD, D/MAX2500, Rigaku, Tokyo, Japan), the component and crystalline phase changes of the geopolymers were examined. The settings were 40 kV of voltage, 30 mA of current, and 0.15418 nm of Cu Kα radiation.

Thermo Fisher Scientific Nicolet IS50 FT-IR (Waltham, MA, USA) analyzer techniques were used to perform Fourier transform infrared spectroscopy (FT-IR) on KBr pellets to determine the phase compositions of the geopolymers. 2.0 cm^−1^ and 16 cm^−1^ were the resolution and scanning times, respectively.

## 3. Results and Discussion

### 3.1. Compressive Strength of Geopolymer

**Figure 2:**
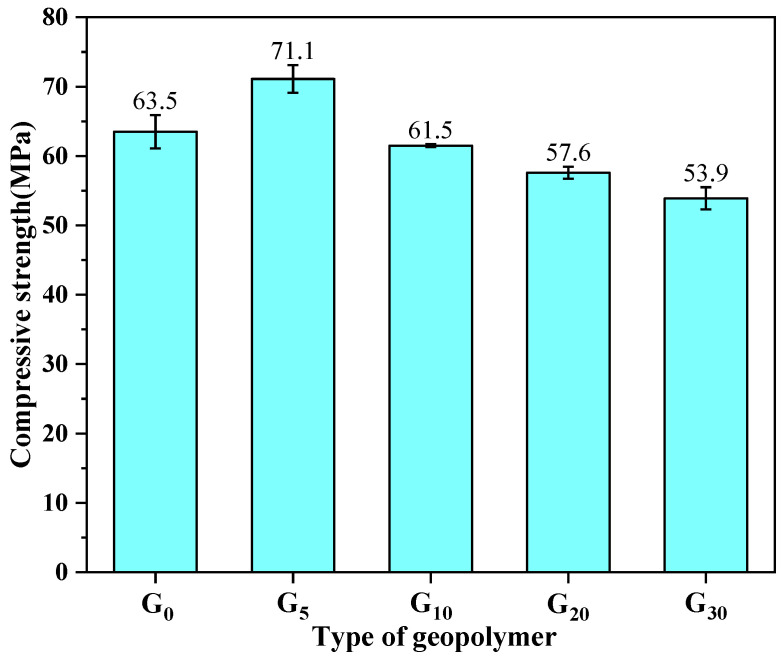
Compressive strength of the geopolymers containing different proportions of CEM I.

**Figure 3:**
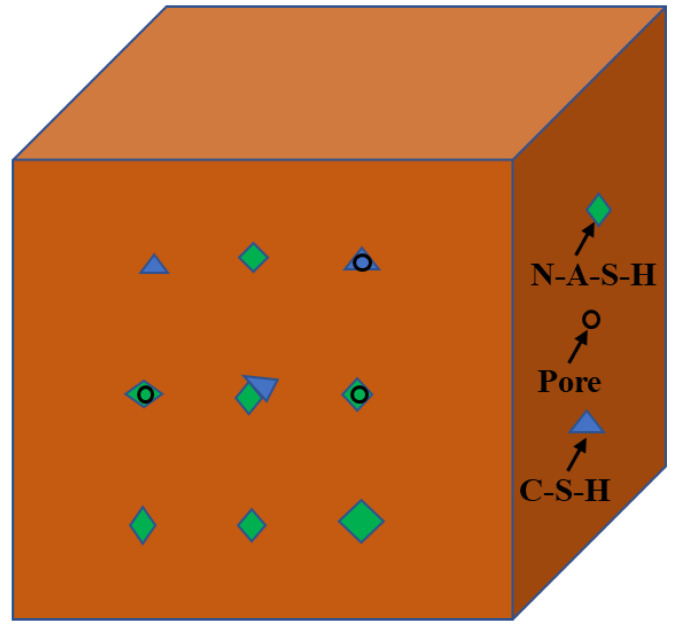
The co-existence of C-S-H gel and N-A-S-H gel in the geopolymers.

The compressive strength of the geopolymers incorporating 0%, 5%, 10%, 20%, and 30% of CEM I is illustrated in Figure 2. As can be seen from the figure, the compressive strength of G_0_, G_5_, G_10_, G_20_, and G_30_ was 63.5 MPa, 71.1 MPa, 61.5 MPa, 57.6 MPa, and 53.9 MPa, respectively. The compressive strength of the geopolymers rose by 12% in comparison to the pure metakaolin-based geopolymer when CEM I was substituted for metakaolin at a rate of 5%. On the one hand, this may be due to the fact that the reaction between CEM I and water in alkali solution was an exothermal process at room temperature, and the heat that was produced to promote the full dissolution of silicon aluminum raw materials, accelerate the process of the geopolymerization reaction, and form a denser three-dimensional network structure, resulting in the enhancement of the compressive strength of the geopolymers [18]. On the other hand, it may be due to the mutual presence of C-S-H gel from the CEM I and N-A-S-H gel from aluminosilicate mix constituents, promoting the more densified microstructure of the geopolymers [25,26], which is presented in Figure 3.

However, as the substitution rate of CEM I increased, the compressive strength of the geopolymers appeared to decline. The compressive strength of G_10_, G_20_, and G_30_ was individually reduced by 3%, 9%, and 15%, in comparison to the specimen of G_0_. From a macro point of view, the decrease in compressive strength was mainly due to the decreased workability of the matrix with regard to using a higher CEM I ratio. From a microscopic point of view, the poor compressive strength development of the aluminosilicate geopolymer incorporating a higher content of cement was due to the fact that the removal of available Si from the solution preferentially by the geopolymerization, which was faster than the hydration of the cement minerals, delayed the formation of C-S-H [27]. Therefore, the optimal substitution rate for CEM I is 5%, at which point the geopolymers have the highest compressive strength.

### 3.2. Flexural Strength of Geopolymers

**Figure 4:**
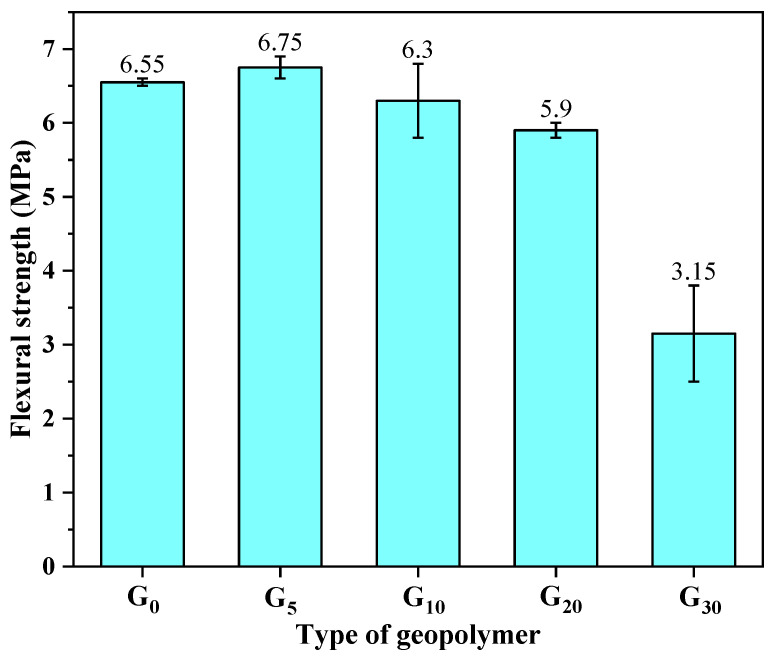
Flexural strength of geopolymers containing different proportions of CEM I.

Figure 4 reveals the flexural strength of the geopolymers incorporated 0%, 5%, 10%, 20%, and 30% of CEM I. Overall, the geopolymers’ flexural strength exhibited an ascending followed by a descending tendency. As a reference group (G_0_), the flexural strength of the geopolymers was 6.55 MPa. With the incorporation of CEM I, the flexural strength of the geopolymers has changed accordingly. Additionally, the flexural strength of G_5_, G_10_, G_20_, and G_30_ was 6.75 MPa, 6.3 MPa, 5.9 MPa, and 3.15 MPa. Compared with the reference group, the flexural strength of G_5_ increased by 3.1%. The presence of an additional calcium source is the reason for the increase in the flexural strength of the geopolymers. The calcium element that was present in the CEM I would react with water to generate extra C-S-H and C-A-S-H, which coexisted with geopolymer products (N-A-S-H) [28] and contributed to the strength growth of the geopolymers.

On the contrary, the flexural strength of G_10_, G_20_, and G_30_ decreased by 3.8%, 9.9%, and 51.9%, respectively, in comparison to the specimen of G_0_. Additionally, the rate of the geopolymers’ flexural strength declined faster as the cement content rose. This was due to the fact that the geopolymer matrix was unable to supply more water for the CEM I hydration reaction, which prevented excessive CEM I from producing hydration products and hindered the growth of the strength of the geopolymers. It was consistent with the results in compressive strength. Therefore, 5% replacement is the ideal rate for the geopolymers to improve their flexural strength.

### 3.3. SEM Analysis

**Figure 5:**
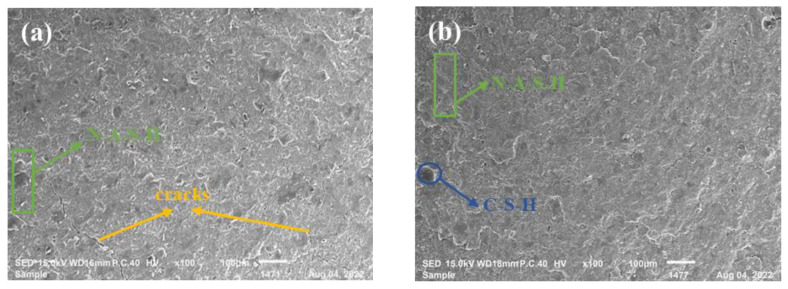

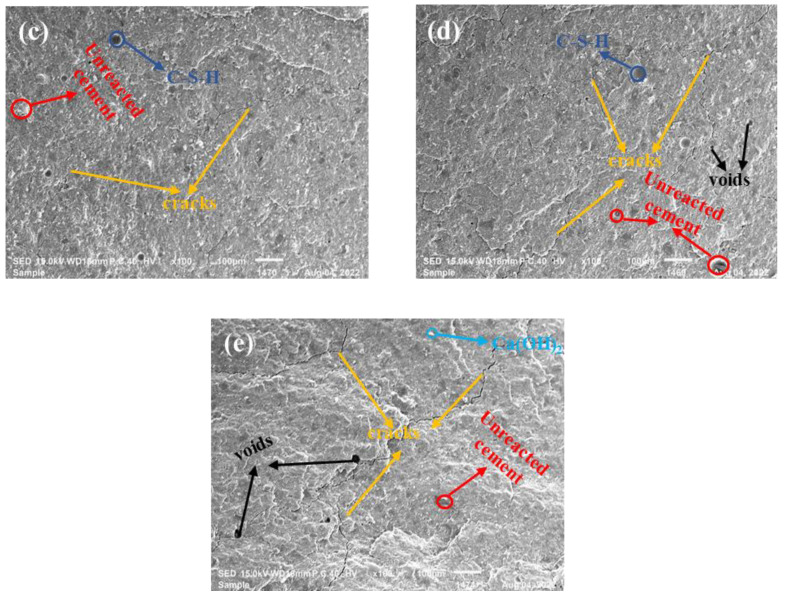
SEM of the geopolymers containing different proportions of CEM I: (**a**) G_0_; (**b**) G_5_; (**c**) G_10_; (**d**) G_20_; and (**e**) G_30_.

Figure 5 presents the micro-morphologies of the geopolymers containing different proportions of CEM I. The microstructure of G_0_ was relatively dense, and only a few microcracks were visible, as can be seen in Figure 5a. Additionally, the geopolymerization of alkali-activated materials resulted in sodium aluminate silicate gels with a strong cross-linked molecular structure formed in the geopolymers, which was the cause of the specimens’ dense internal structure. With the incorporation of CEM I, the hydration reaction of cement also occurred, and the calcium-based hydrated product (C-S-H) was formed in the specimens, as can be seen in Figure 5a–d. The coexistence of C-S-H and the geopolymer product N-A-S-H was found in Figure 5b. Additionally, the coexistence of C-S-H and N-A-S-H could effectively improve the mechanical properties of the specimens [26], consistent with the former results of the mechanical properties.

As the CEM I content incorporated into the geopolymers increased, the density of the microstructure of the geopolymers decreased along with more cracks and voids appearing. In comparison to other geopolymer specimens, the microstructure of G_30_ was unmistakably deteriorated when the substitution rate of CEM I reached 30%. Moreover, the unreacted CEM I particles were also observed in Figure 5c–e. It can be explained that the incomplete hydration reaction of CEM I occurred in the geopolymers. The white precipitate of calcium carbonate (CaCO_3_) was visible in Figure 5e, which was attributed to the reaction between calcium hydroxide (Ca(OH)_2_) and carbon dioxide in the air [29].

### 3.4. XRD Analysis

**Figure 6:**
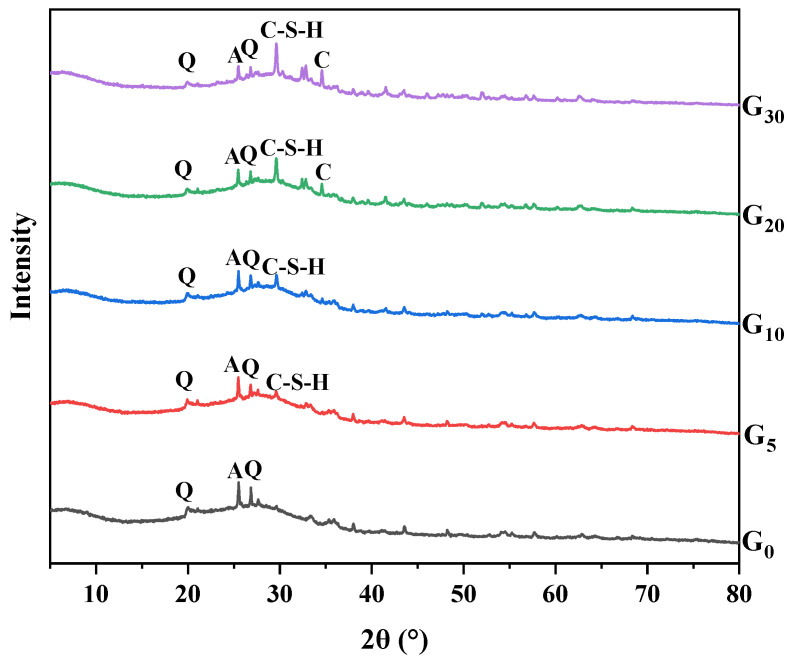
XRD patterns of the geopolymers containing 0% CEM I (G_0_), 5% CEM I (G_5_), 10% CEM I (G_10_), 20% CEM I (G_20_), and 30% CEM I (G_30_). (A: anatase; Q: quartz; C: calcite; and C-S-H: hydrated calcium silicate).

The XRD patterns of the geopolymers containing different proportions of CEM I are revealed in Figure 6. Anatase, quartz, and calcite are the major crystal mineral diffraction peaks in the XRD spectra of the five specimens. As can be seen from the figure, the broad diffuse humps of the geopolymers are at around 2θ = 20°–35°. This is caused by the formation of amorphous aluminosilicate, the main binder phase in the geopolymer matrix, giving the material outstanding mechanical characteristics [30,31]. Comparing the patterns of the different geopolymers, it can be found that though the diffraction peak of quartz could still be detected in the geopolymers, the peak strength decreased gradually with the addition of CEM I. This indicated that the incorporation of CEM I can promote the geopolymerization reaction between silica-alumina materials and alkaline solutions [19]. With more CEM I incorporated into the mixture, the peak strength of anatase decreased, indicating that the content of metakaolin had decreased. The abundant active calcium components existed in CEM I, contributing to the occurrence of the hydration reaction of cement. A significant amount of heat energy was released during this reaction, accelerating the process of geopolymerization and ultimately improving the mechanical characteristics of the geopolymers [32,33]. The geopolymers incorporating cement were found to contain the ordered calcium silicate hydrate (C-S-H), proving that the CEM I’s constituent parts had been dissolved and taken part in the alkali activation reaction.

In addition, the calcite was detected in the spectrum of G_20_ and G_30_. This can be explained by the fact that CEM I has a lot of active calcium components (60.67%); therefore when it was combined with the geopolymer, the water from the alkaline solution would react with the calcium-rich phase to break the Ca-O link and form Ca^2+^ [34]. After that, the Ca(OH)_2_ absorbed the carbon dioxide in the air to produce calcium carbonate, which would negatively affect the structure of the geopolymers and restrict the development of strength. This was consistent with the former analysis.

### 3.5. FT-IR Analysis

**Figure 7:**
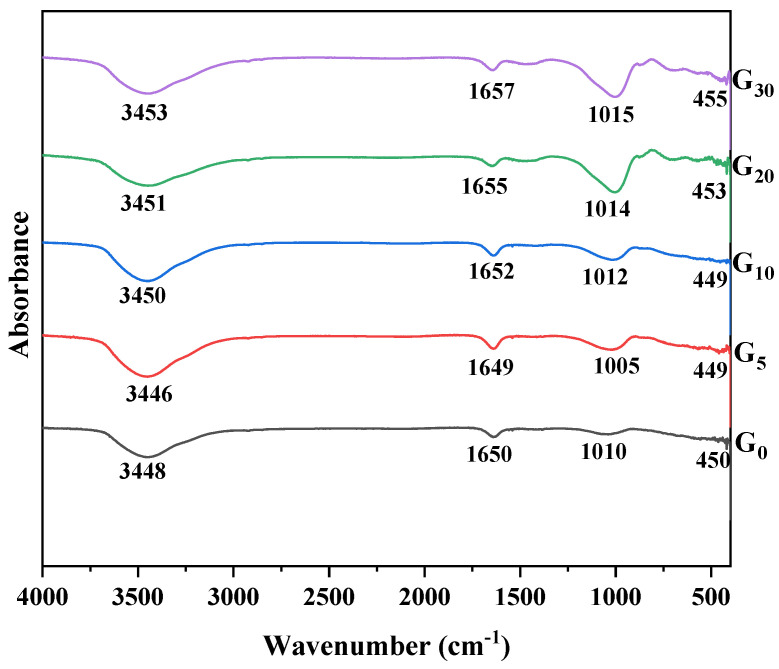
FTIR spectra of geopolymers containing 0% CEM I (G_0_), 5% CEM I (G_5_), 10% CEM I (G_10_), 20% CEM I (G_20_), and 30% CEM I (G_30_).

Figure 7 presents the FTIR spectra of the geopolymers containing different proportions of CEM I. The bands found at 3446–3453 cm^−1^ and 1649–1657 cm^−1^ were attributed to asymmetric/symmetric stretching vibrations of O-H and H-O-H groups in bound water [35], showing that the water molecules were adsorbed on the surface or immersed in pores during geopolymerization [36], according to the results of the FTIR spectra. The band detected at 1005–1015 cm^−1^ was attributed to the tensile vibration of Si-O-T (T = Si or Al). Additionally, it was the consequence of the development of an amorphous N-A-S-H gel network [37]. With the incorporation of CEM I, the spectral band of the geopolymers shifted accordingly. When the substitution rate of CEM I was 5%, the spectral band of the geopolymers moved to higher wavenumbers compared with G_0_, indicating that the gels formed by geopolymerization had obtained a higher degree of cross-linking. However, when more content of CEM I was added to the geopolymers, the spectral bands of the geopolymers moved to lower wavenumbers, manifesting the lower degree of cross-linking of the gels in the geopolymers. The bending vibration of Si-O-Si was assigned based on the band at 449–455 cm^−1^, reflecting the gel phase structure in the geopolymers [38], on account of the fact that the amorphous N-A-S-H gel is the key to ensuring the strength of the geopolymers [39]. Thereby, the 5% content of CEM I was beneficial to improve the strength of the geopolymers, while a larger CEM I incorporation was detrimental to the strength development of the geopolymers. This was consistent with the results obtained in the previous section.

## 4. Conclusions

To clarify the effects of the calcium source on the mechanical properties and microstructures of the geopolymers, CEM I was chosen as the sole calcium source to replace metakaolin at rates of 0%, 5%, 10%, 20%, and 30%. The alkali-activator was a mixture of 12 M NaOH and Na_2_SiO_3_. The compressive strength and flexural strength were used to measure the mechanical properties of the geopolymers. SEM, XRD, and FTIR were used to characterize the microstructure of the geopolymers. The main conclusions can be summarized as follows:

(1) The optimal incorporation of CEM I was 5%, which would endow geopolymer (G_5_) with better mechanical properties and a denser internal structure than the pure metakaolin-based geopolymer (G_0_). The compressive strength and flexural strength of G_5_ can reach 71.1 MPa and 6.75 MPa, respectively.
(2) The heat released by cement hydration can accelerate the process of geopolymerization. Additionally, the co-existence of the hydration reaction product (C-S-H gel) and the geolpolymerization reaction product (N-A-S-H gel) was beneficial to the development of the geopolymers’ strength. This can be explained by the XRD study, which showed that the peak strength gradually dropped after CEM I was added, showing that CEM I can facilitate the geopolymerization process between silica-alumina materials and alkaline solutions.
(3) With the increase in the content of CEM I, the geopolymer matrix was unable to supply more water for the hydration reaction of CEM I, which prevented excessive CEM I from producing hydration products, weakened the workability of the matrix, and eventually hindered the growth of geopolymer strength. In light of the spectra of FT-IR, as more content of CEM I was added into the geopolymers, the spectral bands of the geopolymers moved to lower wavenumbers, indicating the lower degree of cross-linking of gels in the geopolymers.

## Figures and Tables

**Table 1:** Physical properties of metakaolin and CEM I.

Properties	Metakaolin	CEM I
Apparent density (kg/m^3^)	2480	2963
Specific surface area (m^2^/kg)	15,600	350

**Table 2:** Chemical compositions of metakaolin and CEM I (wt%).

Chemical Compositions	Metakaolin	CEM I
SiO_2_	47.69	20.79
Al_2_O_3_	45.10	5.79
Fe_2_O_3_	3.60	2.59
SO_3_	0.19	4.56
TiO_2_	1.95	0.37
CaO	0.58	60.67
K_2_O	0.34	0.72
MgO	0.17	3.86
Na_2_O	0.05	0.20
LOI^a^	0.33	0.45

**Table 3:** Mixed proportion of geopolymers/(kg/m^3^).

Geopolymers	Metakaolin	CEM I	Na_2_SiO_3_	NaOH
G_0_	888	-	500	250
G_5_	843.6	44.4	500	250
G_10_	799.2	88.8	500	250
G_20_	710.4	177.6	500	250
G_30_	621.6	266.4	500	250

## Data Availability

Not applicable.
